# Transcriptomic and metabolomic analyses of three *Dendranthema morifolium* “Boju” varieties with different flower colors

**DOI:** 10.3389/fpls.2025.1690517

**Published:** 2026-02-12

**Authors:** Na Chen, Kai Liu, Gang Li, Rui Liu, Xiao Meng, Wanqiu Zhang, Xuanxuan Guo, Li Zhao, Shengming Ye, Yulei Zhang, Yaowu Liu, Shihai Xing

**Affiliations:** 1 Joint Research Center for Chinese Herbal Medicine of Anhui of Institute of Health and Medicine (IHM), College of Pharmacy, Bozhou Vocational and Technical College, Bozhou, China; 2 Research and Development Department, Bozhou Xinghe Agricultural Development Co, Ltd, Bozhou, China; 3 Marketing Department, Bozhou Wanbei Pharmaceutical Co., Ltd, Bozhou, China; 4 College of Pharmacy, Anhui University of Chinese Medicine, Hefei, China

**Keywords:** *Dendranthema morifolium* “Boju”, differentially expressed genes, flavonoid synthesis, metabolomics, transcriptomics

## Abstract

**Introduction:**

This study aimed to identify the specific genes, key metabolic pathways, and differential metabolites associated with flower color formation in *Dendranthema morifolium* “Boju” (hereinafter, referred to as “Boju”). These analyses were intended to elucidate the causes underlying different flower colors in “Boju” and provide a theoretical basis for optimal breeding of “Boju”.

**Methods:**

“Boju” varieties with pink-white (FB group), yellow-white (HB group), and pure yellow flowers (CH group) were subjected to transcriptomic and metabolomic analyses to identify the differentially expressed genes (DEGs) and differential metabolites in these varieties.

**Results:**

Transcriptomic analysis showed varying enrichment pathways of the DEGs in the three groups. The pathways with higher numbers of enriched genes in the FB vs. CH and FB vs. HB groups were the same, including protein processing in endoplasmic reticulum, plant–pathogen interaction, plant hormone signal transduction, and mitogen-activated protein kinase signal pathway. In the HB vs. CH group, among the four pathways with a larger number of DEGs, three were consistent with the previous two groups. In addition, the number of enriched genes in the starch and sucrose metabolism pathways was also relatively large in this group. Metabolomic analysis indicated that the differential metabolites in the varieties were primarily enriched in the isoflavonoid and flavonoid biosynthesis pathways. In the FB vs. CH, FB vs. HB, and HB vs. CH groups, 28, 29, and 5 differential metabolites were enriched, with most of these metabolites enriched in either isoflavonoid or flavonoid biosynthesis pathways. These findings, combined with real-time quantitative polymerase chain reaction analysis, revealed a significant distribution of the flavonoid synthesis pathway, exceeding the significance threshold line. The differential expression of flavonoid metabolites and genes related to their biosynthetic pathways might primarily impact the formation of different flower colors in “Boju”.

**Discussion:**

Transcriptomic and metabolomic analyses indicated that the types and contents of anthocyanins varied across the “Boju” varieties, resulting in varying flower color. The key differential metabolites were primarily enriched in the isoflavonoid and flavonoid biosynthesis pathways. The DEGs in the different comparison groups exhibited varying enrichment in different pathways, but the enriched pathways were the same across the varieties.

## 1 Introduction

*Dendranthema morifolium* “Boju” is a perennial herb belonging to the Compositae family. Its capitulum is used as medicine. It is one of the medicinal chrysanthemums listed in the 2025 edition of the “Pharmacopoeia of the Peoples Republic of China”. It dispels wind, clears heat, calms the liver, improves eyesight, and detoxifies. The flowers of “Boju” are small and fragrant. It is one of the best qualities of medicinal chrysanthemums. For a long time, “Boju” has been a highly cultivated variety in traditional Chinese medicine in Bozhou city, Anhui Province (Cai et al., 2022).

To date, most of the studies on “Boju” have primarily focused on the verification of materia medica (Liu et al., 2024; Xiong et al., 2025), active components (Wang et al., 2010; Wang et al., 2015), quality control (Li et al., 2015), and pharmacological effects (Chen et al., 2021; Jiang et al., 2021). However, to the best of our knowledge, there have been no reports on the differences in the traits and quality of the three “Boju” varieties with different flower colors. In recent years, with increasing awareness of the advantages of geo-authentic medicinal herbs in medical practice and their growing market demand, research on these herbs has received extensive attention globally. Ouyang et al (Ouyang et al., 2022). studied the differences between two chrysanthemum varieties, “Boju” and “Huaiju”. Peng et al (Peng et al., 2019). extracted the active substances in “Boju” and studied its beneficial effects on hyperuricemia. Based on metabolite characterization, Ling and Guijian (2022) found that “Boju” and “Chuju” were superior among medicinal white chrysanthemum varieties. However, the levels of active ingredients varied across “Boju” varieties with different flower colors. Therefore, elucidating the mechanisms underlying the generation of different flower colors in “Boju” is crucial for understanding the evolution of color variation in this variety and is important for cultivating “Boju” with excellent traits.

Flower color is primarily determined by the types of pigments, such as anthocyanins and carotenoids, in the petals and the flower’s external environment. Most of the structural and regulatory genes in the light-induced anthocyanin pathway are specifically expressed in chrysanthemum (Hong et al., 2016). Anthocyanins are water-soluble natural pigments widely present in plants. They are flavonoid secondary metabolites. Flavonoids confer a wide color range to plants and are an important factor determining the flower color in chrysanthemums (Wang et al., 2021).

In nature, there are at least 20 types of anthocyanins. Among them, common ones include cyanidin, petunidin, peonidin, delphinidin, malvidin, and pelargonidin. The hydroxyl groups of these anthocyanidins combine with different sugars to produce various types of anthocyanins (Song et al., 2021). In this study, we performed comparative transcriptomic and non-targeted metabolomic analyses of three “Boju” varieties with varying flower colors. By analyzing the differentially expressed genes and metabolites in the three varieties, we assessed the mechanisms underlying the generation of varying flower colors, providing a theoretical basis for screening out “Boju” plants with excellent qualities.

## 2 Materials and methods

### 2.1 Experimental materials

The plants used in this experiment were grown in the medicinal plant garden of Bozhou Vocational and Technical College (N 33°46’48”, E 115°48’36”). The samples were identified by Associate Professor Yang Qingshan from Anhui University of Chinese Medicine as a Compositae plant, *Dendranthema morifolium* “Boju”. Voucher specimen was collected and stored at the Herbarium Centre of Anhui University of Traditional Chinese Medicine, Hefei, China. (AhtcmH, yxy.ahtcm.edu.cn/info/1006/6713.htm, Shi-hai Xing, xsh-shihai@163.com, under the No. 20231005).

During the full flowering period, capitula of three colors (**Supplementary Material S1** The capitulum of “Boju” in different colors)—pinkish-white (FB), yellowish-white (HB), and pure yellow (CH)—were collected from the plants that have been transplanted one year after division at 9:00 AM on a sunny morning (20 plants sampled per flower color group, five capitulas were collected per plant, inflorescence diameter: 2.5–5 cm). After sampling, the collected samples were rapidly frozen in liquid nitrogen and stored in a refrigerator at −80°C for use in subsequent metabolomic and transcriptomic analyses.

### 2.2 Experimental methods

#### 2.2.1 Sample preparation, RNA extraction, and transcriptome library construction and sequencing

“Boju” flowers were subjected to non-parametric transcriptome sequencing, with three biological replicates for each treatment. Total RNA was extracted from the three varieties using the TRIzol reagent. After quality check, the RNA was used for the subsequent complementary deoxyribonucleic acid (cDNA) library construction and sequencing. The sequencing platform used was Illumina Novaseq 6000, and the non-parametric transcriptome sequencing was conducted by OE Biotech Co., Ltd. (Shanghai, China).

#### 2.2.2 Data preprocessing, transcriptome assembly, functional annotation, and classification

After data quality control, high-quality clean reads were obtained and assembled into expressed sequence tag clusters (also called contigs). The contigs were then assembled into transcripts from scratch using the Trinity software (Grabherr et al., 2011). Based on sequence similarity and length, the longest transcript was selected as the unigene for subsequent analyses. The functions of unigenes were annotated by comparing them with the National Center for Biotechnology Information non-redundant, Swiss-Prot, and gene evolutionary lineage databases.

These unigenes were also mapped to the Kyoto Encyclopedia of Genes and Genomes (KEGG) for pathway annotation. After annotation, the number of reads aligned to a unigene in each sample was obtained using the bowtie2 software (Langmead and Salzberg, 2012), and the expression level (fragments per kilobase of exon per million mapped fragments (FPKM) value) of each unigene was assessed simultaneously using the express software (Roberts and Pachter, 2013). The DEGs among the sample groups were identified using the DESeq2 software.

During the screening process, the difference multiple was calculated, and the negative binomial distribution test was used to determine the significance of the differences. The condition for screening the differences included q < 0.05. A fold change (FC) of >2 was set as the threshold for determining significantly differentially expressed genes (DEGs). Using the OE Cloud platform, gene ontology (GO) enrichment and KEGG functional annotation were performed on DEGs, and the top 20 genes were selected for enrichment analysis.

#### 2.2.3 DEG screening and reverse transcription quantitative polymerase chain reaction verification

Based on the results of transcriptome sequencing, genes with significant differences in expression levels were screened out and verified by RT-qPCR. Actin was stably expressed in the sample and was selected as an internal reference gene. To ensure the reliability of the results, three biological replicates were analyzed for each experiment. The PCR mix included 2X PerfectStart TM Green qPCR SuperMix, 5μL; 10 μM Forward primer, 0.2 μL;10 μM Reverse primer, 0.2 μL; cDNA, 1 μL; and nuclease-free H_2_O, 3.6 μL. The PCR protocol was as follows: 94°C 30 s, followed by 45 cycles of 94°C 5 s and 60°C 30 s. At the end of the cycle, the product specificity was detected using the melting curve: The temperature was slowly raised from 60°C to 97°C, and fluorescence signals were collected five times at each degree Celsius.

#### 2.2.4 Metabolome determination and analysis

##### 2.2.4.1 Sample preparation

The flowers from the three “Boju” varieties, stored at −80°C, were thawed at room temperature. Six biological replicates were set up for each group. Next, 60 mg of each sample was weighed and added to 1.5-mL EP tubes. Two small steel balls and 600 µL of methanol-water mixture (V:V = 4:1, 4 µg/mL), containing the internal standard, comprising 2-chloro-L-phenylalanine (C2001, Shanghai HC Biotech Co., Ltd, HPLC 98.0%, Shanghai), succinic acid-D4 (293075-1G, Sigma, HPLC98.0%, Shanghai), cholic acid-D4 (S22155-50, Shanghai Yuanye Bio-Technology Co., Ltd. HPLC 98.0%, Shanghai), and D-Luciferin (S19260-100, Shanghai Yuanye BioTechnology Co., Ltd. BR-grade 99.0%, Shanghai), were added to each tube. The purpose of adding internal standards is to test the stability of the instrument. Next, the sample was pre-cooled in a refrigerator at −40°C for 2 min and then ground in a grinding machine (45 Hz, 2 min). The sample was then subjected to ultrasonic extraction in an ice-water bath for 30 min and incubated overnight at −40°C. Next, the sample was centrifuged for 20 min at 12,000 rpm at 4°C. Then, 150 µL of the supernatant was loaded into a liquid chromatography-tandem mass spectrometry (LC-MS/MS) injection vial with an inner tube for analysis. Quality control (QC) samples were prepared by mixing all the samples into a pooled sample (one QC sample per six samples). The relative standard deviation value was controlled within 0.25. All extraction reagents (high-performance liquid chromatography (HPLC)-grade) are pre-cooled at −20°C before use, including methanol (Fisher, A452-4, 99.9%, America), acetonitrile (Fisher, A998-4, 99.95%, America), and methanoic acid (Fisher, A117-50, 99.9%, America).

##### 2.2.4.2 LC-MS/MS analysis

An ACQUITY UPLC I-Class plus (Waters Corporation, Milford, USA) fitted with a Q-Exactive mass spectrometer equipped with a heated electrospray ionization (ESI) source (Thermo Fisher Scientific, Waltham, MA, USA) was used for metabolic profiling in both ESI-positive and -negative ion modes. Chromatography was conducted under the following conditions: Chromatographic column, ACQUITY UPLC HSS T3 (100 mm × 2.1 mm, 1.8 µm); column temperature, 45°C; mobile phases, (A) water (0.1% formic acid-water) and (B) acetonitrile; flow rate, 0.35 mL/min; and injection volume, 4 L. The elution gradients and MS parameters are listed in **Tables 1** and **2**. All the samples were kept at 10°C during the analysis.

**Table 1:** Elution gradient.

Time (min)	Mobile phase A (%)	Mobile phase B (%)
0~2	95	5
2~4	70	30
4~8	50	50
8~10	20	80
10~15	0	100
15~16	95	5

**Table 2:** Mass spectrometry parameters of liquid chromatography–mass spectrometry (LC-MS).

Parameters	Positive ion mode	Negative ion mode
Spray voltage (V)	3800	−3000
Capillary temperature (°C)	320	
Auxiliary gas heater temperature (°C)	350	
Sheath gas flow rate (arb)	35	
Auxiliary gas flow rate (arb)	8	
S-lens RF level	50	
Mass range (*m*/*z*)	70–1050	
Full MS resolution	60,000	
MS/MS resolution	15,000	
NCE/stepped NCE	10, 20, 40	

RF, Radio Frequency; NCE, Normalized Collision Energy.

##### 2.2.4.3 Data preprocessing and statistical analysis

The original LC-MS data were subjected to baseline filtering, peak identification, integration, and retention time (RT) correction by the metabolomics processing software XCMS v4.5.1. The compounds were identified based on multiple dimensions, such as RT, precise mass number, secondary fragments, isotope distribution, precise mass-to-charge ratio (M/Z), secondary fragments, and isotopic distribution using The Human Metabolome Database (HMDB), Lipidmaps (v2.3), Metlin, and Lumet-Plant 3.0 (a self-built database of OE Biotech Co., Ltd.). The differential metabolites were identified based on the following criteria: p-value < 0.05, FC ≥ 4.0 or FC ≤ 1/4. The identified differential metabolites were subjected to KEGG pathway enrichment analysis (http://www.genome.jp/kegg/). The data were recorded using Excel 13.0 and statistically analyzed using SPSS 22.0. p < 0.05 indicated significant differences.

## 3 Results

### 3.1 Transcriptomic analysis of “Boju” flowers with different colors

#### 3.1.1 Sequencing quality analysis

In this study, non-parametric transcriptome sequencing was performed on a total of nine samples from the three “Boju” varieties. Transcriptome sequencing was performed for nine samples. A total of 61.62 G of clean data was obtained, with an effective data volume of each sample distributed between 6.37 G and 7.05 G, the Q30 base distributed between 94.05% and 94.51%, and an average GC content of 42.91% (**Supplementary Material S2** Transcriptome sequencing quality analysis). A total of 70,657 Unigenes were spliced together, with a total length of 72,143,214 bp and an average length of 1,021.03 bp. This result indicated that the quality of RNA-sequencing (RNA-seq) data was high, and it could be used for further analysis.

#### 3.1.2 DEG analysis

**Figure 1:**
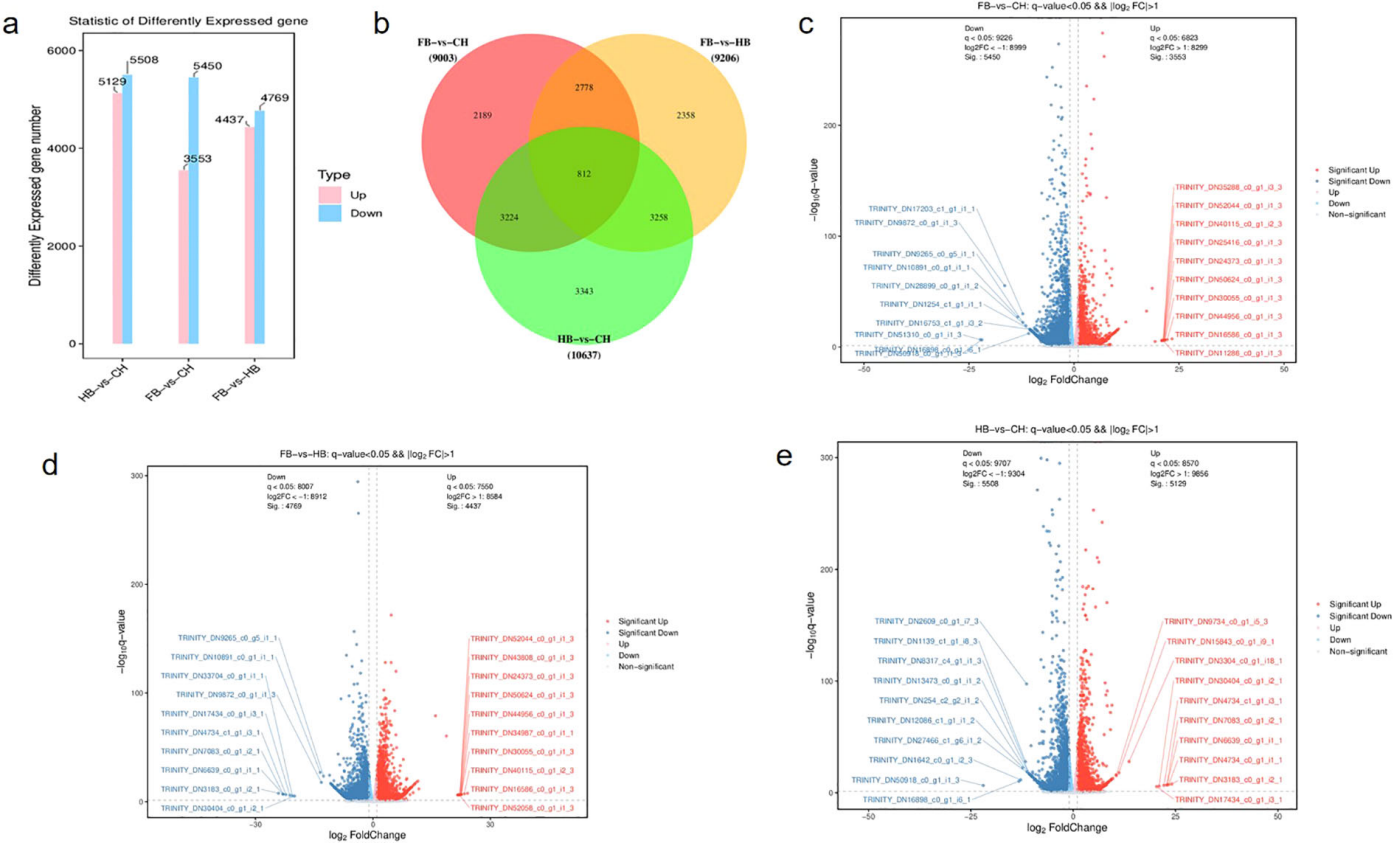
Statistical bar chart of differentially expressed Unigene: Gray represents the non-difference Unigene, red represents the upregulated significant difference Unigene, and blue represents the downregulated significant difference Unigene. The X-axis represents the display of log2FoldChange, and the Y-axis direction represents the display of -log10pValue. **(a)** gene_different stat barplot: The horizontal axis represents each comparison group; The vertical axis represents the number of Unigene differences in the comparison group, Up represents the number of upregulated Unigene differences with significant differences, and Down represents the number of downregulated Unigene differences with significant differences; **(b)** The common and specific differential expressions of Unigene among different comparison groups; **(c)** FB-vs-CH-volcano-q-val-0.05-FC-2.gene; **(d)** FB-vs-HB-volcano-q-val-0.05-FC-2.gene; **(e)** HB-vs-CH-volcano-q-val-0.05-FC-2.gene: The differences resulting from the comparison are reflected in the volcano map.

The DESeq2 software was used to further screen and compare the DEGs among different groups. We detected 10,637 DEGs and 3,343 specific DEGs in HB vs. CH, with 5,129 and 5,508 genes significantly upregulated and downregulated, respectively. In addition, we found 9,003 DEGs and 2,189 specific DEGs in FB vs. CH, with 3,553 and 5,450 genes significantly upregulated and downregulated, respectively. Moreover, we identified 9,206 DEGs and 2,358 specific DEGs in FB vs. HB, with 4,437 and 4,739 genes significantly upregulated and downregulated, respectively. Among all three comparison groups, we detected a total of 812 DEGs (**Figures 1a, b**). The overall distribution of differentially expressed genes in each comparison group was shown in the differentially expressed volcano plot (**Figures 1c–e**).

#### 3.1.3 Functional annotation of DEGs

To elucidate the metabolic pathways related to different flower colors in “Boju”, GO annotation analysis was performed on the DEGs of each comparison group, mainly annotating them to biological processes (BP), cell components and molecular function (MF).

**Figure 2:**
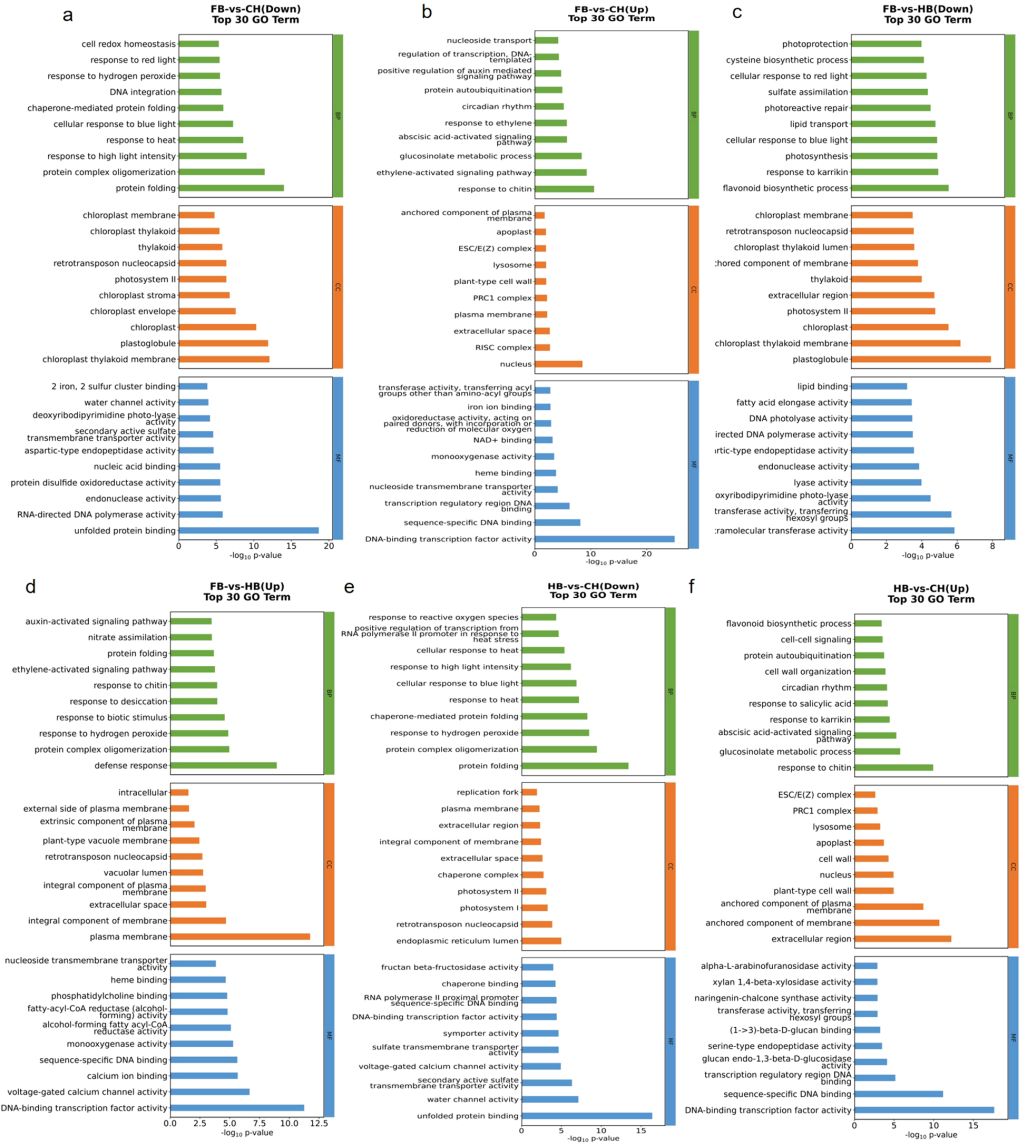
Top30 GO Enrichment Analysis (Screen the GO entries in the three categories that correspond to PopHits ≥ 5, and sort 10 entries for each category in descending order of their corresponding -log10PValue) **(a)** GO top. Down of FB vs CH; **(b)** GO top. UP of FB vs CH; **(c)** GO top. Down of FB vs HB; **(d)** GO top. UP of FB vs HB; **(e)** GO top. down of HB vs CH; **(f)** GO top. UP of HB vs CH.

In the FB vs. CH group, the upregulated DEGs were mainly annotated to responses to chitin, nucleus, and DNA-binding transcription factor activity. The downregulated DEGs were annotated to protein folding, plasmoglobin, chloroplast thylakoid membrane, and unfolded protein binding (**Figures 2a, b**).

In the FB vs. HB group, the upregulated DEGs were primarily annotated to defense response, plasma membrane, DNA-binding transcription factor activity, etc. The downregulated DEGs were annotated to flavonoid biosynthetic process, plastoglobule, and transferase activity (**Figures 2c, d**).

In the HB vs. CH group, the upregulated DEGs were mainly annotated to the response to chitin, plasma membrane-anchored components, extracellular regions, and DNA-binding transcription factor activity. The downregulated DEGs were mainly annotated to protein folding, endoplasmic reticulum lumen, and unfolded protein binding, among others (**Figures 2e, f**).

Thus, the upregulation of DNA-binding transcription factor activity was found to be a critical MF characteristic shared among the three comparison groups. Moreover, both the FB vs. CH and HB vs. CH groups exhibited a downregulation of protein folding-related functions. The differences among the three comparison groups were primarily reflected in the biosynthesis of flavonoids and the activities of related enzymes.

#### 3.1.4 Enrichment analysis of KEGG signaling pathways

**Figure 3:**
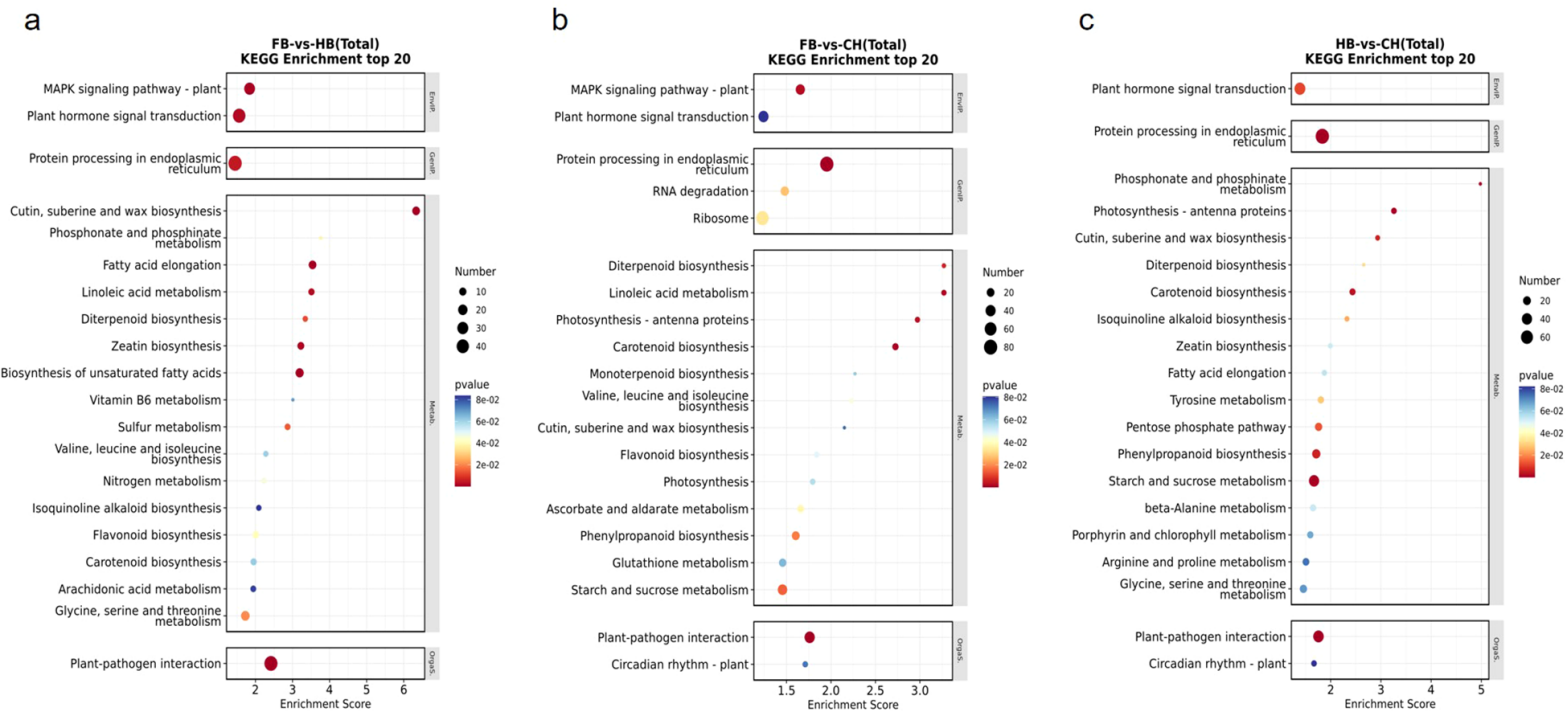
**(a)** FB vs HB (total) KEGG Enrichment top 20; **(b)** FB vs CH (total) KEGG Enrichment top 20; **(c)** HB vs CH (total) KEGG Enrichment top 20. In the figure, the horizontal axis “Enrichment Score” represents the enrichment score. The larger the bubble, the greater the number of differential Unigenes contained in the entry. The bubble color changes from blue to white, yellow to red. The smaller the enrichment p-value, the greater the significance.

Pathway analysis of the differential unigenes was performed using the KEGG database. Our results indicated that among the top 20 pathways, the pathways with a larger number of enriched genes in the two comparison groups, FB vs. CH and FB vs. HB, were consistent, including protein processing in the endoplasmic reticulum, plant–pathogen interaction, plant hormone signal transduction, and mitogen-activated protein kinase signal pathway-plant (**Figures 3a, b**). In the HB vs. CH group, three of the four pathways with a higher number of DEGs were consistent with the other two comparison groups, namely plant hormone signal transduction, protein processing in the endoplasmic reticulum, and plant–pathogen interaction. The number of genes enriched in the starch and sucrose metabolic pathways in the HB vs. CH group was also relatively large (**Figure 3c**).

In the FB vs. CH group (**Figure 3b**), linoleic acid metabolism, photosynthesis antenna proteins, and carotenoid biosynthesis were significantly enriched with high enrichment scores. Although the plant hormone signal transduction pathway contained a relatively large number of target genes, its enrichment significance was low. In the HB vs. CH group (**Figure 3c**), the phosphonate and phosphinate metabolism pathway exhibited the highest enrichment score; however, the number of DEGs enriched in this pathway was relatively small.

Overall, plant hormone signal transduction, protein processing in the endoplasmic reticulum, and plant–pathogen interaction were the core differential pathways enriched among the three comparison groups. This result indicated that these BP were common and critical among the three comparison groups. However, each comparison group exhibited unique changes in the aggregation pathway.

#### 3.1.5 Verification of key DEGs using SYBR GREEN RT-PCR

**Figure 4:**
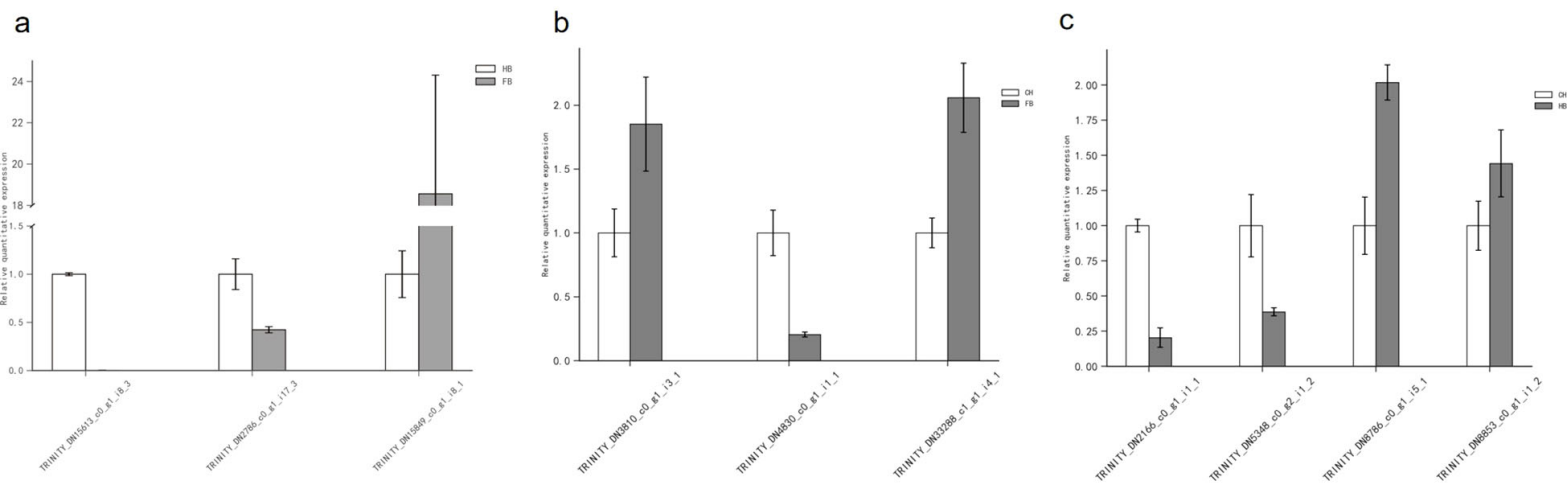
SYBR GREEN fluorescence quantitative PCR verification of differentially expressed genes X-axis: Differentially expressed genes, Y:-axis:relative quantitative expression. **(a)** The expression levels of the differentially expressed genes in the HB vs FB, **(b)** The expression levels of the differentially expressed genes in the CH vs FB, **(c)** The expression levels of the differentially expressed genes in the CH vs HB.

Based on the results of the enrichment analysis, ten DEGs (**Table 3**) were screened out and verified by SYBR GREEN RT-PCR. The PCR results are shown in **Figure 4**. The results of RNA-seq were consistent with those of SYBR GREEN RT-PCR, indicating that the transcriptome data were accurate and reliable.

**Table 3:** Primer list.

Gene symbol	Forward primer (5->3)	Reverse primer (5->3)	Product length (bp)	Tm (°C)
TRINITY_DN10059_c0_g2_i2_2 (Actin)	CTCATAAGACAAAGCTGACTG	TGGAGGAGGATAGACATCTG	89	60
TRINITY_DN4830_c0_g1_i1_1	ATCAACCGCGTTAAGGATG	ATCTTCCCTGAAGCAATATCG	92	60
TRINITY_DN33288_c1_g1_i4_1	CACCAGTGATGCTGGACA	ACTACTACCATCATCGCTCT	89	60
TRINITY_DN15613_c0_g1_i8_3	TCGCGGTATTAGAGAACACG	ACTCTTGAGGCGATACTTTG	106	60
TRINITY_DN2786_c0_g1_i17_3	CATCTTCAAGGAGAAGCGG	TTATCAGGCCACCTGGAGT	95	60
TRINITY_DN15849_c0_g1_i8_1	GACAGCAGCATGTGCATTA	AAGCTGCGTCAATAGTCG	83	60
TRINITY_DN2166_c0_g1_i1_1	CTTGTGACAAGTGTGGCTC	ACAAAGGTAGCACACACATT	91	60
TRINITY_DN5348_c0_g2_i1_2	GTTGTCAGAGTCTGCGAG	CCGTAACTATCGGTCCCT	84	60
TRINITY_DN8786_c0_g1_i5_1	CATGAGAGAACTTCCTGTGG	TCATCAGTAACCAACACATGC	87	60
TRINITY_DN8853_c0_g1_i1_2	TAGAGAAGGCACTGACACAT	ATCATCAAGTATTGCTGGACC	101	60

### 3.2 Metabolomics analysis

#### 3.2.1 Analysis of anthocyanin components in the “Boju” varieties

We determined the types of anthocyanins and the relative contents of their components in the “Boju” varieties (**Table 4**). We detected six anthocyanins in all three varieties, namely cyanidin, petunidin, peonidin, delphinidin, malvidin, and pelargonidin. Among them, the relative contents of cyanidin and peonidin in FB were higher than those in HB and CH, with the levels of most components of cyanidin significantly higher in FB than in HB and CH. In contrast, the relative content of malvidin in FB was significantly lower than that in HB and CH. Moreover, the relative contents of most components of delphinidin and malvidin differed significantly among all three varieties.

**Table 4:** The anthocyanin fractions and relative contents in *D. morifolium* “Boju” varieties.

Anthocyanin class	Components	Relative content (μg/g)		
		FB	HB	CH
Cyanidin	Cyanidin-3-O-galactoside	27.586 ± 0.243a	24.873 ± 0.757b	24.705 ± 0.771b
	Cyanidin-3-o-beta-glucopyranoside	21.540 ± 0.110a	19.475 ± 0.397b	19.233 ± 0.231b
	Cyanidin 3-(3’’,6’’-dimalonylglucoside)	22.734 ± 0.186a	20.162 ± 0.331b	20.217 ± 0.287b
	Cyanidin 3,5-di-(6-malonylglucoside)	24.223 ± 0.276a	21.556 ± 0.293b	20.672 ± 0.489b
	Cyanidin 3,5-di-(6-acetylglucoside)	22.181 ± 0.112a	21.748 ± 0.151b	21.135 ± 0.080c
	Cyanidin 3-(6’’-malonyl-2’’-glucuronosylglucoside)	22.167 ± 0.233a	21.699 ± 0.306ab	20.935 ± 0.484b
	Cyanidin 3,5-diglucoside (6’’,6’’’-malyl diester)	20.543 ± 0.143a	20.295 ± 0.325a	19.477 ± 0.527a
Petunidin	Petunidin	17.332 ± 1.340a	19.330 ± 0.178a	18.873 ± 0.174a
Peonidin	Peonidin	24.966 ± 0.204a	24.021 ± 0.765a	23.935 ± 0.846a
Delphinidin	Delphinidin	18.293 ± 0.121b	19.197 ± 0.327a	19.027 ± 0.197a
	Delphinidin 3-arabinoside	19.576 ± 0.181b	20.313 ± 0.148a	20.026 ± 0.119ab
	Leucodelphinidin	20.311 ± 0.269a	19.500 ± 0.219a	20.226 ± 0.289a
Malvidin	Malvidin	22.844 ± 0.251b	24.660 ± 0.244a	24.071 ± 0.278a
Pelargonidin	Pelargonidin 3-(6’’-succinylglucoside)-5-glucoside	23.459 ± 0.377a	22.240 ± 0.215b	22.272 ± 0.174b
	Leucopelargonidin 3-O-alpha-L-rhamno-beta-D-glucopyranoside	17.003 ± 1.441b	20.168 ± 0.285a	19.618 ± 0.288a
	pelargonidin-3-O-rutinoside	18.226 ± 0.805a	19.582 ± 0.410a	19.315 ± 0.675a
	Pelargonidin 3-arabinoside	17.610 ± 1.417a	19.445 ± 0.329a	17.159 ± 1.330 a

Different lowercase letters in the same column indicate significant differences between groups at p < 0.05; the same letters indicate no significant difference.

#### 3.2.2 Analysis of differential metabolites in the three “Boju” varieties

Partial least squares (PLS) regression was used to establish a relationship model between the expression levels of metabolites and the sample grouping to determine the differential metabolites in the three “Boju” varieties.

**Figure 5:**
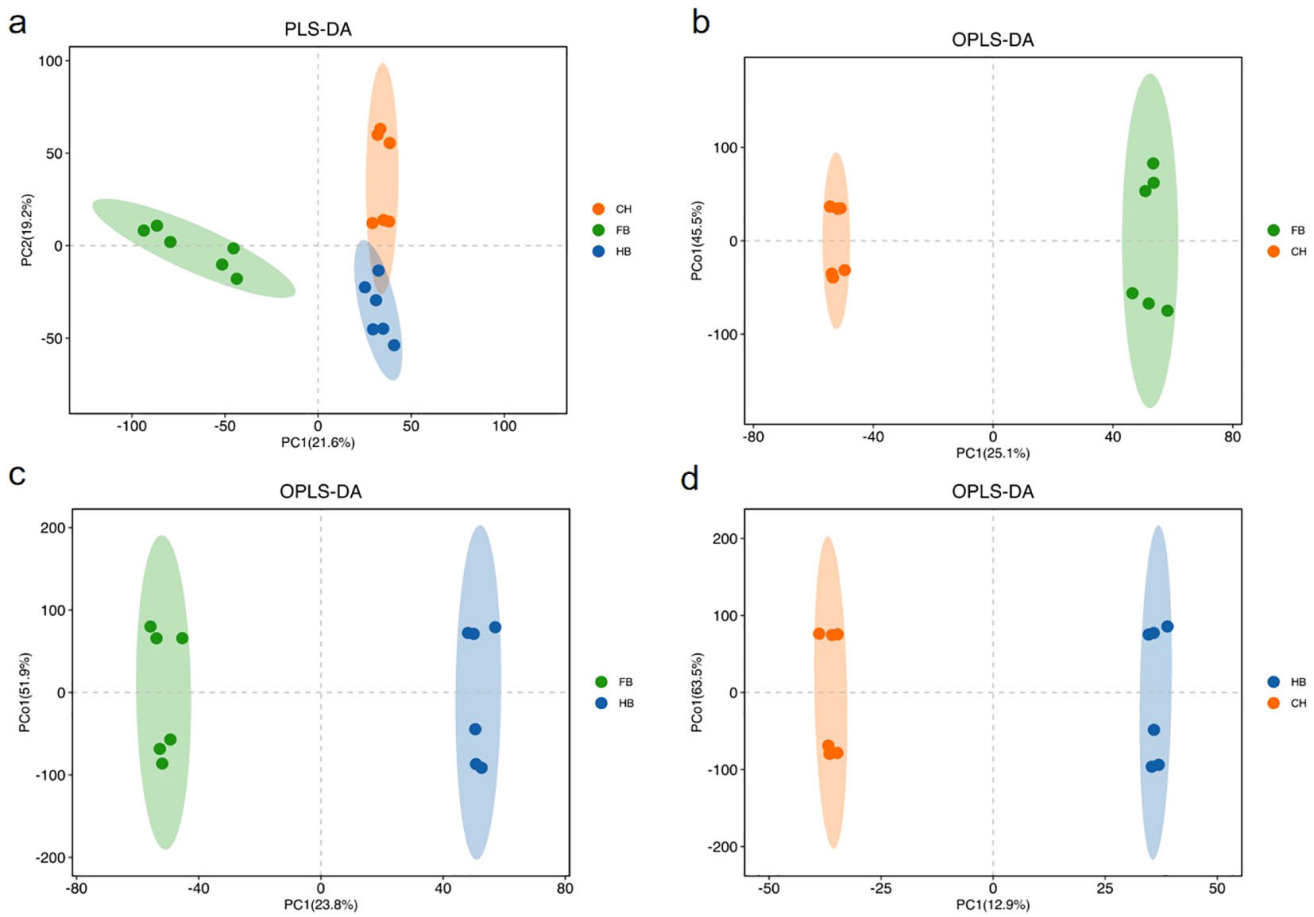
**(a)** PLS-DA score group All sample; **(b)** OPLS-DA score FB-vs-CH; **(c)** OPLS-DA score FB-vs-HB; **(d)** OPLS-DA score HB-vs-CH. OPLS-DA maximizes the inter-group differences reflected on t1, so the inter-group variations can be directly identified from t1, while the intra-group variations are reflected on the orthogonal principal components.

Through PLS-discriminant analysis (DA), the three groups of samples were well grouped. Based on PLS-DA, modifications were made, and orthogonal PLS-DA (OPLS-DA) was adopted to maximize the reflection of the differences among the three groups (**Figure 5a**). Every two groups of samples exhibited significant differences on the OPLS-DA score graph (**Figures 5b–d**).

**Figure 6:**
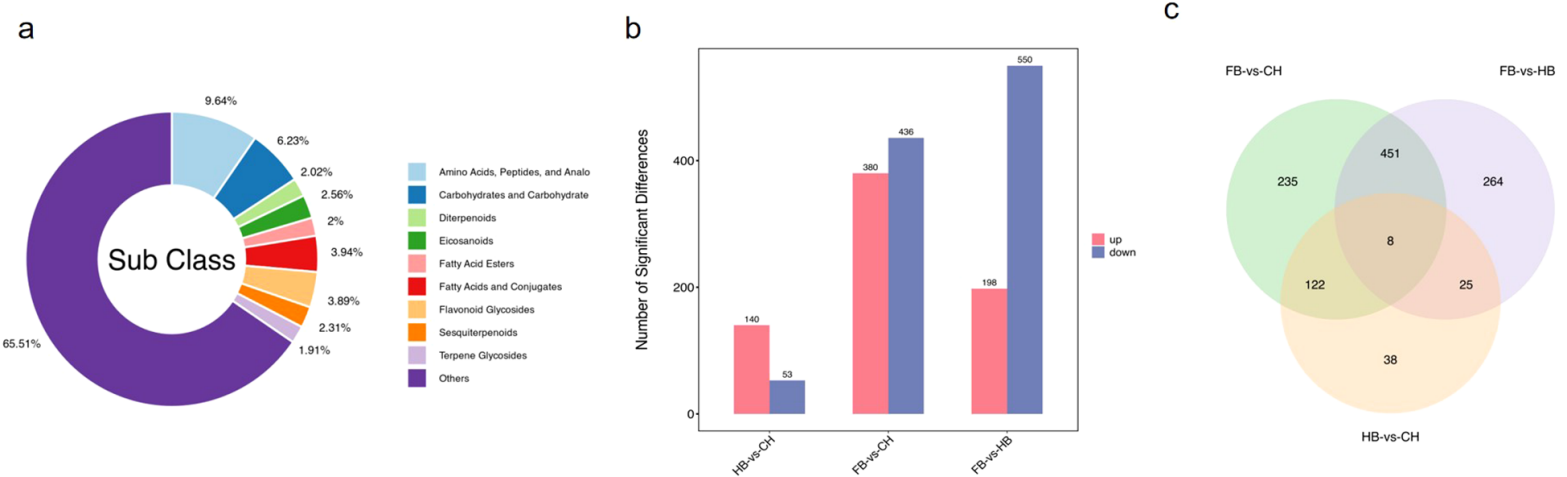
Statistical analysis of differentially expressed metabolites. **(a)** Material sub classification statistics pie chart; **(b)** Bar chart of the difference in the quantity of substances in each group; **(c)** Venn diagrams of the common and specific differential metabolites in the difference comparison group.

We detected a total of 8,762 metabolites. The differential metabolites were classified and statistically analyzed by substance classification. Amino acids, peptides, and analogs accounted for 9.64%, carbohydrates and conjugates accounted for 6.23%, fatty acids and conjugates accounted for 3.94%, and flavonoid glycosides accounted for 3.88% (**Figure 6a**). Using p-value < 0.05 and FC ≥ 4.0 as the screening criteria, the differential metabolites among the three differential groups were statistically compared. We detected 816 differential metabolites in the FB vs. CH group, with 380 upregulated and 436 downregulated metabolites. We found 748 differential metabolites in the FB vs. HB group, with 198 upregulated and 550 downregulated metabolites. In addition, we identified 193 differential metabolites in the HB vs. CH group, with 140 upregulated and 53 downregulated metabolites (**Figures 6b, c**).

**Figure 7:**
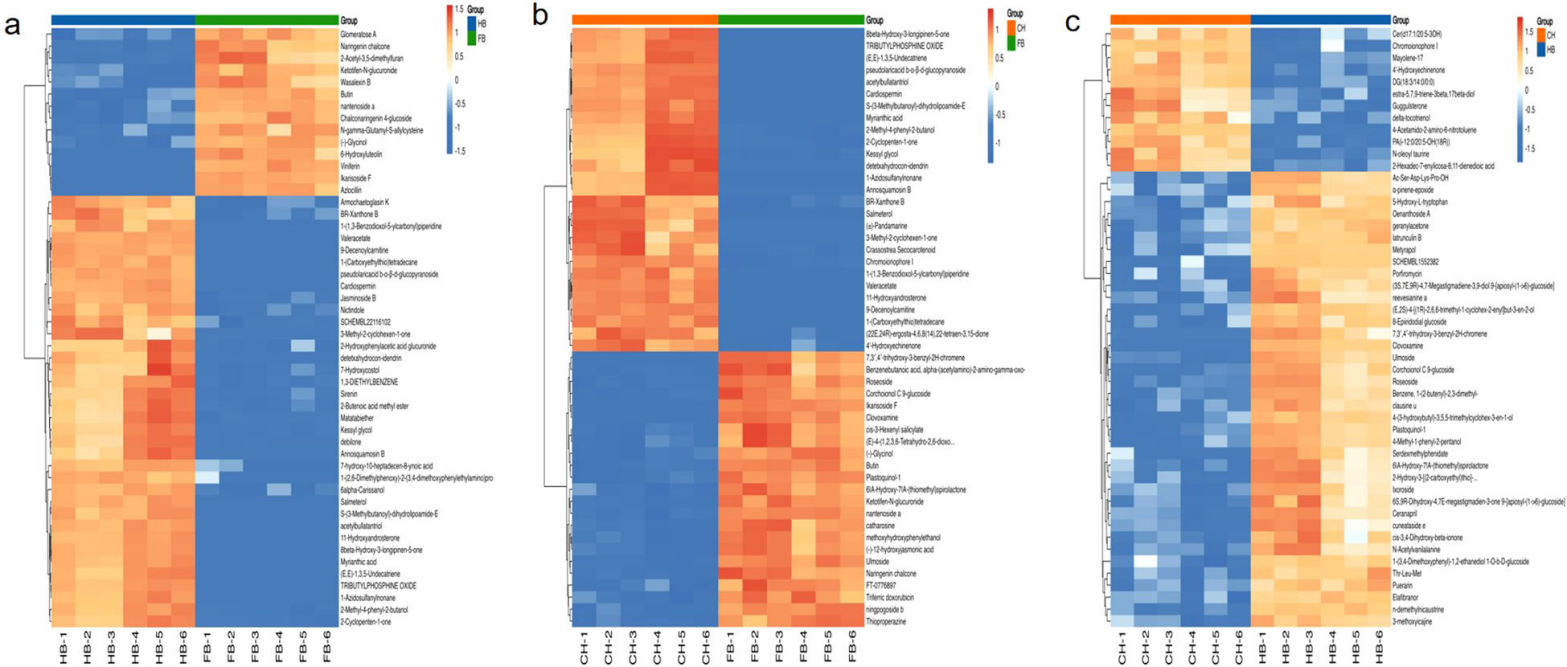
Hierarchical clustering analysis of the expression levels of differential metabolites **(a)** Hierarchical Clustering analysis of the expression levels of differential metabolites in FB vs BH; **(b)** Hierarchical Clustering analysis of the expression levels of differential metabolites in FB vs CH; **(c)** Hierarchical Clustering analysis of the expression levels of differential metabolites in HB vs CH.

To more intuitively demonstrate the relationship and expression differences of metabolites among different comparison groups, hierarchical clustering analysis was performed on the expression levels of all differential metabolites and the differential metabolites with a minimum p-value of Top50. The hierarchical clustering diagram showed that in the FB vs. CH group, butin, naringenin chalcone, methoxy hydroxyphenylethanol, etc., were more abundant in FB, while annosquamosin B, 4’-hydroxyechinenone, etc., were more abundant in CH. In the FB vs. HB group, butin, naringenin chalcone, 6-hydroxyluteolin, chalconaringenin 4-glucoside, and ikarisoside F were more abundant in FB (**Figures 7a, b**). In the HB vs. CH group, 4’-hydroxyechinenone, chromoionophore I, etc., were more abundant in CH, while puerarin, puerarin, and 6S, 9R-dihydroxy-4, 7E-megastigmadien-3-one 9-[apiosyl- (1–6)-glucoside] were more abundant in HB (**Figure 7c**).

#### 3.2.3 Analysis of differential metabolic pathways in the “Boju” varieties

KEGG pathway enrichment analyses were conducted on all differentially expressed metabolites, including those that were upregulated and downregulated.

**Figure 8:**
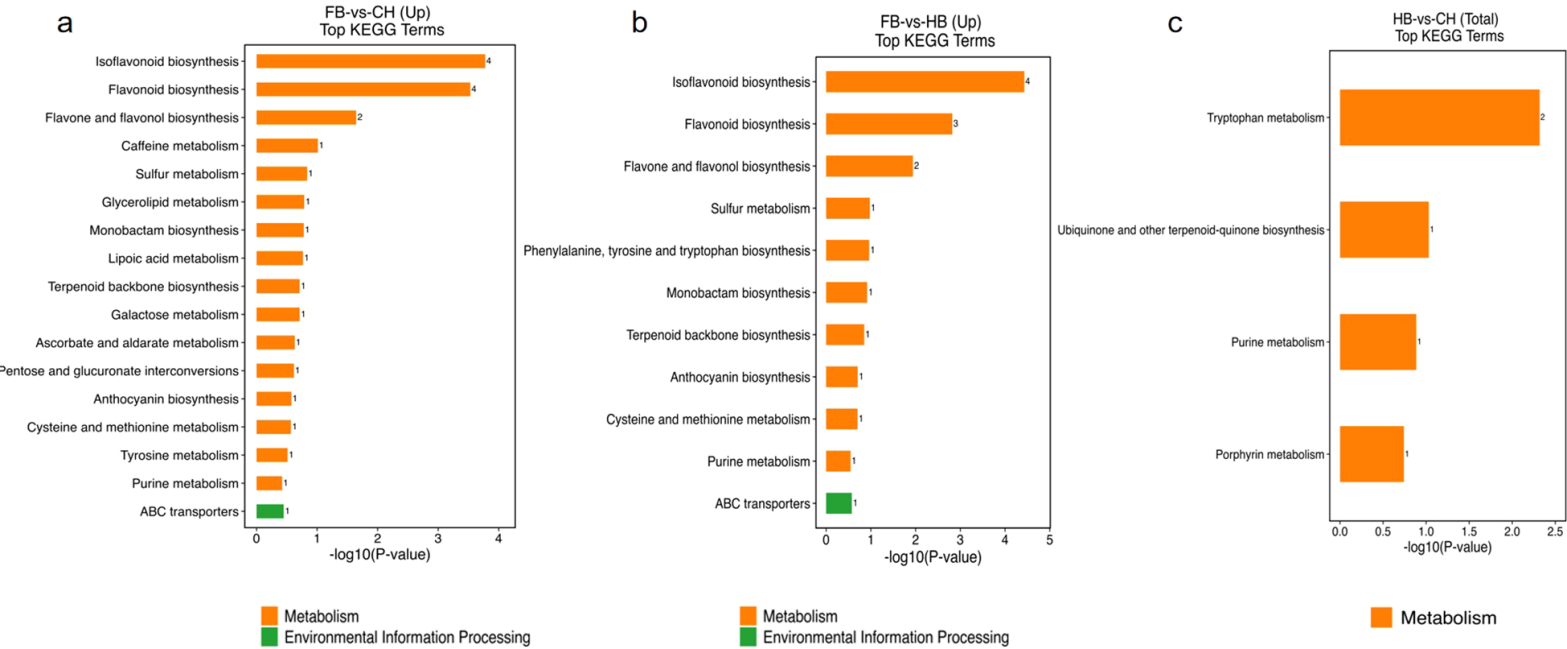
Statistics of metabolic pathways. **(a)** Top 20 KEGG Terms in FB vs CH(up); **(b)** Top 20 KEGG Terms in FB vs HB (up); **(c)** Top 20 KEGG Terms in HB vs CH (Total).

In the FB vs. CH group, 28 differential metabolites were enriched in the KEGG pathways. Four of the upregulated differential metabolites—dihydroquercetin, naringenin, naringenin chalcone, and butin—were enriched in two pathways, isoflavonoid biosynthesis and flavonoid biosynthesis (**Figure 8a**). In the FB vs. HB group, 29 differential metabolites were enriched in the KEGG pathways (**Figure 8b**). Among the upregulated differential metabolites, naringenin, 6-hydroxydaidzein, malonylgenistin, and 3, 6, 9-trihydroxypterocarpan were enriched in the isoflavonoid biosynthesis pathway (**Supplementary Material S3** han943 Isolavonoid biosynthesis). In addition, naringenin, butin, and naringenin chalcone were enriched in flavonoid biosynthetic pathways (**Supplementary Material S4** han941 Flavonoid biosynthesis). In the HB vs. CH group, a total of five differential metabolites were enriched and annotated into four KEGG pathways. Among them, two upregulated differential metabolites were enriched in the tryptophan metabolism pathway, but not in the isoflavone or flavonoid biosynthesis pathways (**Figure 8c**). The differential metabolites related to isoflavones and flavonoids were relatively similar between the HB and CH groups. These differential metabolites were primarily enriched in two pathways, the isoflavonoid biosynthesis pathway and the flavonoid biosynthesis pathway, including anthocyanin biosynthesis.

### 3.3 Integrated transcriptome and metabolome analysis

**Figure 9:**
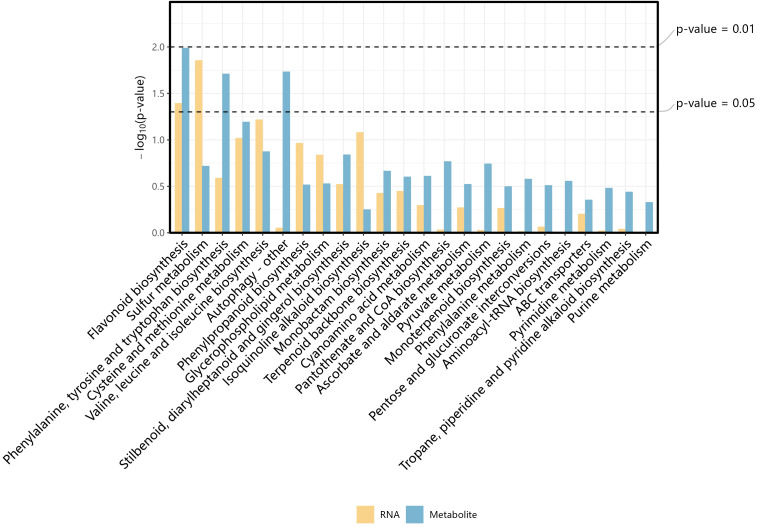
Analysis of the top 30 pathways enriched in metabolomic and transcriptomic analyses in FB-vs-HB. Draw bar charts for the top30 common pathways in each group and mark the significance threshold lines, X-axis: represents the name of the KEGG pathway; Y:-axis: represents the enrichment p-value of the KEGG pathway in each group.

Analysis of the top 30 pathways enriched in metabolomic and transcriptomic analyses revealed that the flavonoid synthesis pathway was significantly distributed in both omics and exceeded the significance threshold line (**Figure 9**). The value of the significance threshold is p < 0.05. In the FB vs. HB group, the differential metabolites and DEGs were enriched in the flavonoid biosynthetic pathway. Metabolomic analysis indicated that both the flavonoid and anthocyanin biosynthesis pathways showed significant enrichment and overall upregulation of metabolites. The genes related to the flavonoid biosynthesis pathway showed a downregulation trend, which might contribute to the differences in flower color among these varieties (**Supplementary Material S5** ko00941 Flavonoid biosynthesis). In the FB vs. CH group, metabolomic analysis revealed no significant enrichment of the phenylpropanoid biosynthesis pathway, indicating that there were no substantial differences in the levels of metabolites related to this pathway. However, the phenylalanine, tyrosine, and tryptophan biosynthesis pathways were significantly enriched, with the related metabolites showing a downregulation trend. Experimental data showed that compared with the CH group, the expression level of the ANS gene (TRINITY_DN4830_c0_g1_i1_1) in the FB group was significantly decreased (**Figure 4**), while the accumulation of peonidin was significantly increased (**Table 4**). This reverse change relationship indicates that the ANS gene is a negative regulatory factor in the biosynthesis pathway of peonidin. In the HB vs. CH group, the combined transcriptome and metabolome analysis did not reveal significant co-enrichment of any of the metabolic pathways, which might be attributed to the insignificant differences in metabolite levels between the two varieties.

**Figure 10:**
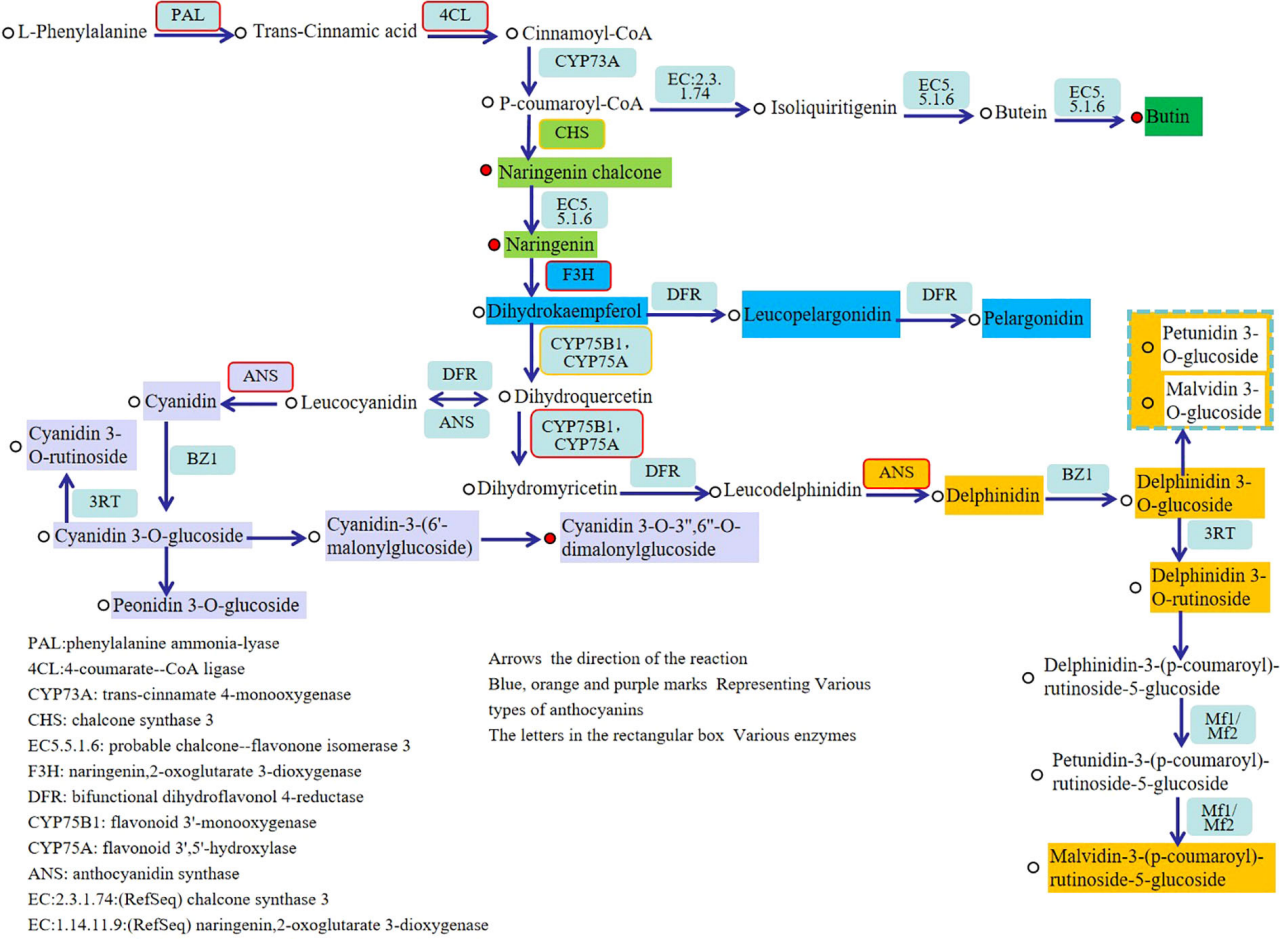
The synthetic pathway of anthocyanins: Differentially expressed genes and metabolites in different colors to visually illustrate regulatory relationships.

By analyzing combined metabolomics and transcriptomics data, the biosynthesis pathways of different anthocyanins in “Boju” were constructed (**Figure 10**). We detected that phenylalanine is deaminated under the catalysis of phenylalanine ammonia-lyase to generate trans-cinnamic acid. Trans-cinnamic acid is then combined with coenzyme A to form cinnamoyl-CoA, which then undergoes a hydroxylation reaction to produce p-coumaroyl-CoA, which serves as a key intermediate metabolite in the phenylalanine metabolic pathway. Naringenin chalcone is biosynthesized using p-coumaroyl-CoA as the precursor under the catalysis of chalcone synthase (*CHS*).

Subsequently, naringenin chalcone is isomerized into naringenin under the action of chalcone isomerase. Naringenin is converted into dihydrokaempferol under the catalysis of flavanone 3-hydroxylase (*F3H*). Dihydrokaempferol is an important precursor for anthocyanin synthesis, generating leucopelargonidin, leucocyanidin, and leucodelphinidin under the catalysis of dihydroflavonol 4-reductase. These colorless anthocyanidins are further oxidized to form pelargonidin, cyanidin, and delphinidin under the action of anthocyanidin synthase (*ANS*).

In addition, p-coumaroyl-CoA also generates butin via another branch of metabolic pathways. In the subsequent modifications of anthocyanins, cyanidin can be methylated to form peonidin, and delphinidin can be further methylated to form petunidin and malvidin. This series of reactions plays a crucial role in the synthesis of plant anthocyanins and the regulation of flower color.

## 4 Discussion

“Boju”, one of the “four famous medicinal chrysanthemums,” is primarily cultivated through division or cuttings due to seed abortion. After long-term asexual reproduction, the yield and quality of “Boju” have been decreasing year by year, which has attracted the attention of many researchers. A previous study indicated that the number of flowers per plant is the main factor contributing to the yield of medicinal chrysanthemums (Huang et al., 2024). Significant differences have been detected in the contents of chlorogenic acid, caffeic acid, and linarin among samples from different regions (Guo et al., 2010). Previously, Li et al. indicated that differences in color and chemical composition affect the quality of chrysanthemums, which might be attributed to variations in chlorogenic acid levels (Li et al., 2022).

In the current study, we conducted metabolomic and transcriptomic analyses of three “Boju” varieties with different flower colors and found significant differences in the levels of metabolites and DEGs among them. Venn diagram analysis revealed 812 DEGs and eight differential metabolites among the three comparison groups. In the HB vs. CH group, 10,637 DEGs (5,129 upregulated and 5,508 downregulated) and 193 differential metabolites (140 upregulated and 53 downregulated) were detected. In the FB vs. CH group, 9,003 DEGs (3,553 upregulated and 5,450 downregulated) and 816 differential metabolites (380 upregulated and 436 downregulated) were detected. In the FB vs. HB group, 9,206 DEGs (4,437 upregulated and 4,769 downregulated) and 748 differential metabolites (198 upregulated and 550 downregulated) were detected.

Anthocyanins are natural pigments derived from the phenylpropanoid pathway. They are synthesized through secondary metabolism in higher plants via the flavonoid branch of the phenylpropanoid pathway. They are widely present in flowers, fruits, seeds, leaves, and stems of higher plants, imparting different colors to these parts (Ge et al., 2025). Currently, anthocyanin biosynthetic pathways and related genes are being extensively studied. Studies have suggested that the cyclization of lycopene is a key link in determining flower color. The modification of the related TFs will break the expression balance between the upstream and downstream genes and greatly influence the carotenoid profile in flowers (Zhang et al., 2024), and Deng’s found that both anthocyanins and carotenoids contribute significantly to the formation of floral color that determines the color signal, and the upregulation of structural genes of CHS, F3’H, DFR and ANS on the anthocyanin biosynthesis pathway in petals was identified, as well as three genes of LCYE, BCH, and CCD4 on the carotenoid biosynthesis pathway (Deng et al., 2023). In the HB vs. CH group, the number of genes enriched in the starch and sucrose metabolic pathways was also relatively large. Starch and sucrose metabolism pathways play crucial roles in Aquilegia Salt Stress Adaption (Chen et al., 2023). By the gene interaction analysis that AGPase and SBE were core genes for the starch and sucrose metabolism pathway in Taro Corm (Dong et al., 2021).

From the phenotypic perspective, the color of FB flowers was lighter. TRINITY_DN4830_c0_g1_i1_1, namely the *ANS* gene, which usually encodes a key enzyme in the anthocyanin synthesis pathway (Yin et al., 2025), catalyzes the conversion of colorless anthocyanins into colored ones. Transcriptome analysis revealed that *ANS* was downregulated in the FB group compared to the CH group, resulting in inhibition of the biosynthesis of anthocyanins, ultimately resulting in lighter flower color. Studies have shown that *ANS* levels positively correlate with the depth of flower color (Feng et al., 2023), which is consistent with the results of the current study. In addition to gene-mediated regulation, biochemical pathways and environmental factors within plants (Li et al., 2017) also impact changes in flower color.

The anthocyanin synthesis process involves multiple catalytic enzymes. Besides *ANS* (Jaakola, 2013), the synthesis of the upstream metabolites of the anthocyanin biosynthesis process, such as dihydrokaempferol, naringenin chalcone, and butin, is also regulated by other enzymes, such as F3H, *CHS, CYP75A*, and *CmMYB* (Yin et al., 2021). Previously, Geng et al (Geng et al., 2024). showed that *CmMYB*308 is a key regulatory factor for the pink color in “Danding” chrysanthemums. Wang et al (Wang et al., 2022). reported that *CmMYB9a* is involved in the regulation of flavonoid-related pathways in chrysanthemums. Han et al (Han et al., 2012). showed that the expression of key structural genes, such as *CHS*, *CHI*, *F3H*, *F3’H*, *DFR*, and *ANS*, resulted in the accumulation of cyanidin in ray florets of chrysanthemum. Transcriptome sequencing revealed a significant *CHS* upregulation, accompanied by significantly enhanced levels of its catalytic product, naringenin chalcone. This finding indicated that *CHS* expression might directly promote the biosynthesis of this metabolite.

Furthermore, *F3H* levels directly affect flavonoid synthesis and regulate the accumulation of secondary metabolites in plants (Jing et al., 2021). When this gene is highly expressed, the catalysis of dihydroquercetin generation is enhanced, promoting the synthesis of anthocyanins and deepening the color of the flowers. In the FB vs. HB group, *F3H* was downregulated, resulting in reduced dihydrokaempferol generation. Moreover, dihydroquercetin is produced under the regulation of flavonoid 3’-monooxygenase (*CYP75B1*) and flavonoid 3’,5’-hydroxylase (*CYP75A*), which potentially reduces anthocyanin synthesis and leads to variations in pink flower color.

Studies have shown that peonidin and cyanidin are purplish-red, while geraniums appear brick-red (Liu et al., 2024). The appearance, color, and depth of the peanut seed coat are mainly impacted by the levels of cyanidin glycosides and delphinidin glycosides.

The color of the seed coat deepens with increasing levels of these two glycosides (Su et al., 2022). The composition and content of carotenoids can greatly influence the color phenotype of plants (Zhang et al., 2024), and research has indicated that upregulated carotenoid concentrations and carotenoid biosynthesis-related genes predominantly promote color transition (Xia et al., 2021).

In addition to cyanidin and peonidin, delphinidin and petunidin also impact the depth of purple-red color. These compounds play an important role in the synthesis of anthocyanins and synergistically regulate the expression of plant colors. The results of the present study indicated that the “Boju” flower color is primarily associated with the types and contents of cyanidin glycosides and peonidin glycosides. Cyanidin glycosides, delphinidin glycosides, flavonoids and flavonol metabolites contribute greatly to the color of “Black Cat”, with quercetin glycoside and isorhamnetin glycoside crucially affecting the color of Den-Phals (Lin et al., 2025). The difference in the flower color of the FB group from that of the other two varieties might primarily be attributed to relatively higher contents of peonidin and cyanidin in FB.

Thus, under the activity of multiple anthocyanins, peonidin and cyanidin play a crucial role in the generation of color in FB. The differences in the plant flower colors are closely related to the synthesis of anthocyanins (Wu et al., 2023). Although cyanidin glycosides are the main coloring substances in purple-red vegetables, the specific types and contents of cyanidin compounds vary across species and varieties. These differences might be attributed to genetic differences, and further research and exploration are needed in this context.

## Data Availability

The data has been uploaded to the public platform. link: https://pan.baidu.com/s/15X2hr6SPiY5tDC-X81lPfA?pwd=agkj

## Abbreviations

ANS: Anthocyanidin synthase
BP: Biological processes
CC: Cell components
CHS: Chalcone synthase
DA: Discriminant analysis
DEGs: Differentially expressed genes
ESI: Electrospray ionization
FC: Fold change
GO: Gene ontology
KEGG: Kyoto Encyclopedia of Genes and Genomes
MF: Molecular function
NB: Negative binomial
NR: Non-redundant
PLS: Partial least squares
QC: Quality control
RT: Retention time
SCAR: Sequence-Characterized Amplified Region
FB: *Dendranthema morifolium* ‘Boju’.with pinkish-white capitulum
HB: *Dendranthema morifolium* ‘Boju’.with yellowish-white capitulum
CH: *Dendranthema morifolium* ‘Boju’.with pure yellow capitulum
DNA: Deoxyribonucleic Acid
RNA: Ribonucleic Acid
RSD: Relative Standard Deviation
HPLC: High Performance Liquid Chromatography
LC-MS/MS: Liquid chromatography-tandem mass spectrometry
CYP75B1: flavonoid 3’-monooxygenase
CYP75A: flavonoid 3’,5’-hydroxylase.

## References

[B1] Cai Y. Gao Y. Zhang Z. Liu H. Wang Y. Ma Y. . (2022). Development and application of a Cultivar-Specific Sequence-Characterized Amplified Region (SCAR) marker for the detection of *Chrysanthemum morifolium* Ramat. 'Daboju'. Plants. 11, 604. doi: 10.3390/plants11050604, PMID: 35270074 PMC8912837

[B2] Chen L. Liu Y. Huang X. Zhu Y. Li J. Miao Y. . (2021). Comparison of chemical constituents and pharmacological effects of different varieties of *Chrysanthemum flos* in China. Chem. Biodivers. 18, e2100206. doi: 10.1002/cbdv.202100206, PMID: 34142430

[B3] Chen L. Meng Y. Bai Y. Yu H. Qian Y. Zhang D. . (2023). Starch and sucrose metabolism and plant hormone signaling pathways play crucial roles in aquilegia salt stress adaption. Int. J. Mol. Sci. 24, 3948. doi: 10.3390/ijms24043948, PMID: 36835360 PMC9966690

[B4] Deng X. Hu C. Xie C. Lu A. Luo Y. Peng T. . (2023). Metabolomic and transcriptomic analysis reveal the role of metabolites and genes in modulating flower color of paphiopedilum micranthum. Plants (Basel). 12, 2058. doi: 10.3390/plants12102058, PMID: 37653975 PMC10220555

[B5] Dong W. He F. Jiang H. Liu L. Qiu Z. (2021). Comparative transcriptome sequencing of taro corm development with a focus on the starch and sucrose metabolism pathway. Front. Genet. 12, 771081. doi: 10.3389/fgene.2021.771081, PMID: 34858484 PMC8630585

[B6] Feng Y. Yang S. Li W. Mao J. Chen B. Ma Z. (2023). Genome-wide identification and expression analysis of ANS family in strawberry fruits at different coloring stages. Int. J. Mol. Sci. 24, 125–154. doi: 10.3390/ijms241612554, PMID: 37628740 PMC10454780

[B7] Ge X. Wu J. Yao Q. Dou S. Li H. (2025). Research progress on flower color and leaf color of ornamental crabapple. Mol. Plant Breed. 04, 1–9. doi: 10.48130/opr00250025

[B8] Geng Z. Liu M. Wang Y. Wang Y. Wang Y. Sun Y. . (2024). Transcriptomic and metabolomic analyses reveal CmMYB308 as a key regulator in the pink flower color variation of 'Dante Purple' chrysanthemum. Plant Cell Rep. 43, 157. doi: 10.1007/s00299-024-03244-5, PMID: 38819475

[B9] Grabherr M. G. Haas B. J. Yassour M. Levin J. Z. Thompson D. A. Amit I. . (2011). Trinity: reconstructing a full-length transcriptome without a genome from RNA-Seq data. Nat. Biotechnol. 29, 644–652. doi: 10.1038/nbt.1883, PMID: 21572440 PMC3571712

[B10] Guo Q. S. Fang H. L. Shen H. J. (2010). Determination of chlorogenic acid, caffeic acid and linarin in Flos Chrysanthemi Indici from different places by RP-hPLC. China J. Chin. Mater Med. 35, 1160–1163. doi: 10.4268/cjcmm20100917, PMID: 20707074

[B11] Han K. Zhao L. Tang X. Hu K. Dai S. (2012). The relationship between the expression of key genes in anthocyanin biosynthesis and the color of chrysanthemum. Acta Hortic. Sinica. 39, 516–524. doi: 10.16420/j.issn.0513-353x.2012.03.015

[B12] Hong Y. Yang L. Li M. Dai S. (2016). Comparative analyses of light-induced anthocyanin accumulation and gene expression between the ray florets and leaves in chrysanthemum. Plant Physiol. Biochem. 03, 120–132. doi: 10.1016/j.plaphy.2016.03.006, PMID: 26990403

[B13] Huang Z. Ci H. Liu Z. Xue Y. Ren X. Xue J. . (2024). Comprehensive evaluation on yield and quality of medicinal *Chrysanthemum morifolium* carieties cased on principal component analysis and cluster analysis. Sci. Technol. Food Ind. 45, 271–280. doi: 10.13386/j.issn1002-0306.2023050062

[B14] Jaakola L. (2013). New insights into the regulation of anthocyanin biosynthesis in fruits. Trends Plant Sci. 18, 477–483. doi: 10.1016/j.tplants.2013.06.003, PMID: 23870661

[B15] Jiang S. Wang M. Jiang Z. Zafar S. Xie Q. Yang Y. . (2021). Chemistry and pharmacological activity of sesquiterpenoids from the chrysanthemum genus. Molecules. 26, 30–38. doi: 10.3390/molecules26103038, PMID: 34069700 PMC8161347

[B16] Jing Z. H. Yin M. Wang Q. Bao K. Zhou P. Liu C. . (2021). Expression profiling and functional verification of flavonoid 3′-hydroxylase gene from leaves of Euryale ferox. China J. Chin. Mater Med. 46, 4712–4720. doi: 10.19540/j.cnki.cjcmm.20210614.101, PMID: 34581080

[B17] Langmead B. Salzberg S. L. (2012). Fast gapped-read alignment with Bowtie 2. Nat. Methods 9, 357–359. doi: 10.1038/nmeth.1923, PMID: 22388286 PMC3322381

[B18] Li H. Qiu J. Chen X. Hong Y. Liang X. (2017). Differential expression of genes associated with anthocyanin synthesis in peanut cultivars with different testa color. Chin. J. Oil Crop Sci. 39, 600–605. doi: 10.7717/peerj.3776/fig8

[B19] Li J. Han Z. Chi L. Wei M. Ye Z. Wu M. . (2022). Germplasm resource evaluation of *Chrysanthemi Indici Flos* based on color and chemical components. China J. Chin. Mater Med. 47, 5217–5223. doi: 10.19540/j.cnki.cjcmm.20220616.102, PMID: 36472028

[B20] Li Y. Wang S. Zhu J. Wang W. Xiang S. Feng W. . (2015). Study on effects of sulfur fumigation on chemical constituents of *Chrysanthemum morifolium* cv. Boju. China J. Chin. Mater Med. 40, 2624–2628. doi: 10.4268/cjcmm20151325, PMID: 26697689

[B21] Lin F. Y. Lu S. J. Liao Y. (2025). Differential analysis of flavonoids metabolites in Den-phals with different floral color based on extensively targeted metabolomics. Mol. Plant Breeding. 11, 1–14. doi: 10.7717/peerj.2870/table-2

[B22] Ling Z. Guijian L. (2022). Analysis of inorganic element content characteristics in different varieties of medicinal chrysanthemum. Chin. Traditional Patent Med. 44, 2414–2417. doi: 10.7287/peerj.15328v0.1/reviews/1

[B23] Liu R. Kou Y. Chen Q. Niu P. Wang X. Ge H. . (2024). Detection of anthocyanins in Rosa plants and the effect on petal color. J. Plant Genet. Resour. 25, 800–812. doi: 10.1016/j.jep.2024.118198, PMID: 38621465

[B24] Liu Y. Lu C. Zhou J. Zhou F. Gui A. Chu H. . (2024). *Chrysanthemum morifolium* as a traditional herb: a review of historical development, classification, phytochemistry, pharmacology and application. J. Ethnopharmacol. 10, 1181–1198. doi: 10.1016/j.jep.2024.118198, PMID: 38621465

[B25] Ouyang H. Fan Y. Wei S. Chang Y. He J. (2022). Study on the chemical profile of chrysanthemum (*Chrysanthemum morifolium*) and the evaluation of the similarities and differences between different cultivars. Chem. Biodivers. 19, e202200252. doi: 10.1002/cbdv.202200252, PMID: 35831709

[B26] Peng A. Lin L. Zhao M. Sun B. (2019). Identifying mechanisms underlying the amelioration effect of *Chrysanthemum morifolium* Ramat. 'Boju' extract on hyperuricemia using biochemical characterization and UPLC-ESI-QTOF/MS-based metabolomics. Food Funct. 10, 8042–8055. doi: 10.1039/C9FO01821B, PMID: 31746890

[B27] Roberts A. Pachter L. (2013). Streaming fragment assignment for real-time analysis of sequencing experiments. Nat. Methods 10, 71–73. doi: 10.1038/nmeth.2251, PMID: 23160280 PMC3880119

[B28] Song J. H. Guo C. K. Shi M. (2021). Anthocyanin biosynthesis and transcriptional regulation in plant. Mol. Plant Breed. 19, 3612–3620. doi: 10.1021/acs.jafc.0c02460.s001

[B29] Su Q. Jin X. Li Y. Cheng Z. Song Y. Yang Y. . (2022). Analysis of key metabolites and *ANS* genes affecting seed Testa color of peanut. Acta Agric. Boreali-Occidentalis Sin. 37, 19–25. doi: 10.3390/bpqn2013-01168

[B30] Wang T. Shen X. Guo Q. Zhou J. Mao P. Shen Z. (2015). Comparison of major bioactive components from leaves of Chrysanthemum morifolium. China J. Chin. Mater Med. 40, 1670–1675. doi: 10.4268/cjcmm20150908, PMID: 26323127

[B31] Wang Y. Guo Q. Shao Q. Zhang Z. (2010). Effects of soil factors on active component content of Chrysanthemum morifolium. China J. Chin. Mater Med. 35, 676–681. doi: 10.4268/cjcmm20100603, PMID: 20545185

[B32] Wang Y. Zhou L. Wang Y. Liu S. Geng Z. Song A. . (2021). Functional identification of a flavone synthase and a flavonol synthase genes affecting flower color formation in Chrysanthemum morifolium. Plant Physiol. Biochem. 07, 1109–1120. doi: 10.1016/j.plaphy.2021.07.019, PMID: 34328869

[B33] Wang Y. Zhou L. Wang Y. Geng Z. Liu S. Chen C. . (2022). CmMYB9a activates floral coloration by positively regulating anthocyanin biosynthesis in chrysanthemum. Plant Mol. Biol. 108, 51–63. doi: 10.1007/s11103-021-01206-z, PMID: 34714494

[B34] Wu D. Zhuang F. Wang J. Gao R. Zhang Q. Wang X. . (2023). Metabolomics and transcriptomics revealed a comprehensive understanding of the biochemical and genetic mechanisms underlying the color variations in chrysanthemums. Metabolites. 13, 742. doi: 10.3390/metabo13060742, PMID: 37367900 PMC10301146

[B35] Xia Y. Chen W. Xiang W. Wang D. Xue B. Liu X. . (2021). Integrated metabolic profiling and transcriptome analysis of pigment accumulation in *Lonicera japonica* flower petals during colour-transition. BMC Plant Biol. 21, 98. doi: 10.1186/s12870-021-02877-y, PMID: 33596836 PMC7890969

[B36] Xiong S. Xie J. Xiang F. Yu J. Li Y. Xia B. . (2025). Research progress on pharmacological effects against liver and eye diseases of flavonoids present in *Chrysanthum indicum* L., *Chrysanthemum morifolium* Ramat., *Buddleja officinalis* Maxim. and *Sophora japonica* L. J. Ethnopharmacol 2, 1190–1194. doi: 10.1016/j.jep.2024.119094, PMID: 39532220

[B37] Yin X. Guan R. Zhang C. Zhao Y. Ran Z. Zhao Q. . (2025). Cloning and expression analysis of ANS gene in three flower colors of Scutellaria baicalensis. Shan Dong Agric. Sci. 57, 19–26. doi: 10.14083/j.issn.1001-4942.2025.03.003

[B38] Yin X. J. Zhang Y. B. Zhang L. Wang B. Zhao Y. Irfan M. . (2021). Regulation of MYB transcription factors of anthocyanin synthesis in lily flowers. Front. Plant Sci. 12, 761–768. doi: 10.3389/fpls.2021.761668, PMID: 34925411 PMC8672200

[B39] Zhang M. Chai Z. H. Zhang C. Chen L. (2024). Unbalanced expression of structural genes in carotenoid pathway contributes to the flower color formation of the osmanthus cultivar 'Yanzhi hong'. Int. J. Mol. Sci. 25, 10198. doi: 10.3390/ijms251810198, PMID: 39337681 PMC11432492

