# Supplementary figures and images for: Transcriptomic and metabolomic analyses of three Dendranthema morifolium “Boju” varieties with different flower colors
Source: Front Plant Sci. 2026 Feb 12;16:1690517. doi: 10.3389/fpls.2025.1690517 (PMC12935939; doi:10.3389/fpls.2025.1690517)

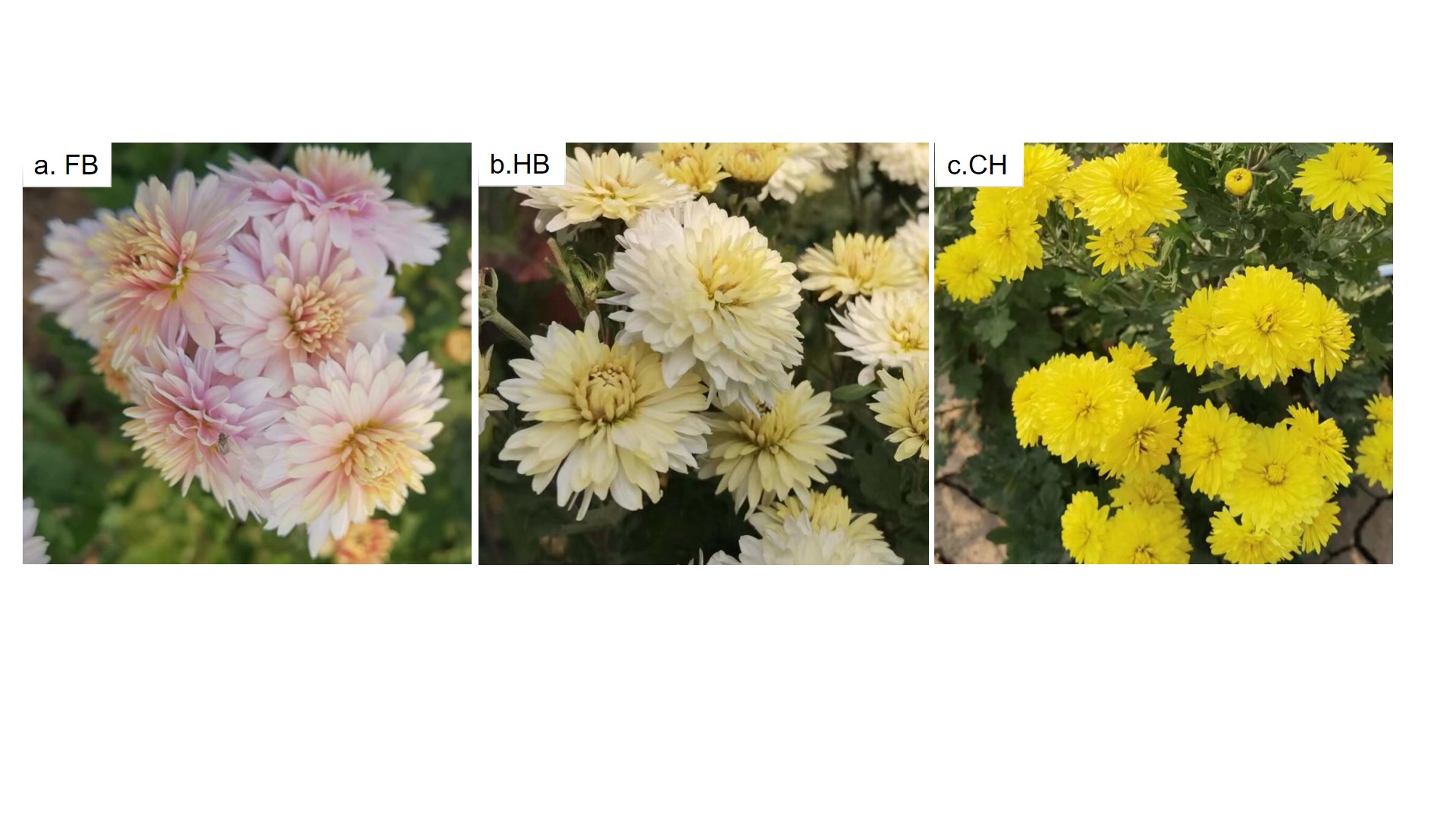

Supplement: Supplementary file 1 [file Image1.jpeg]

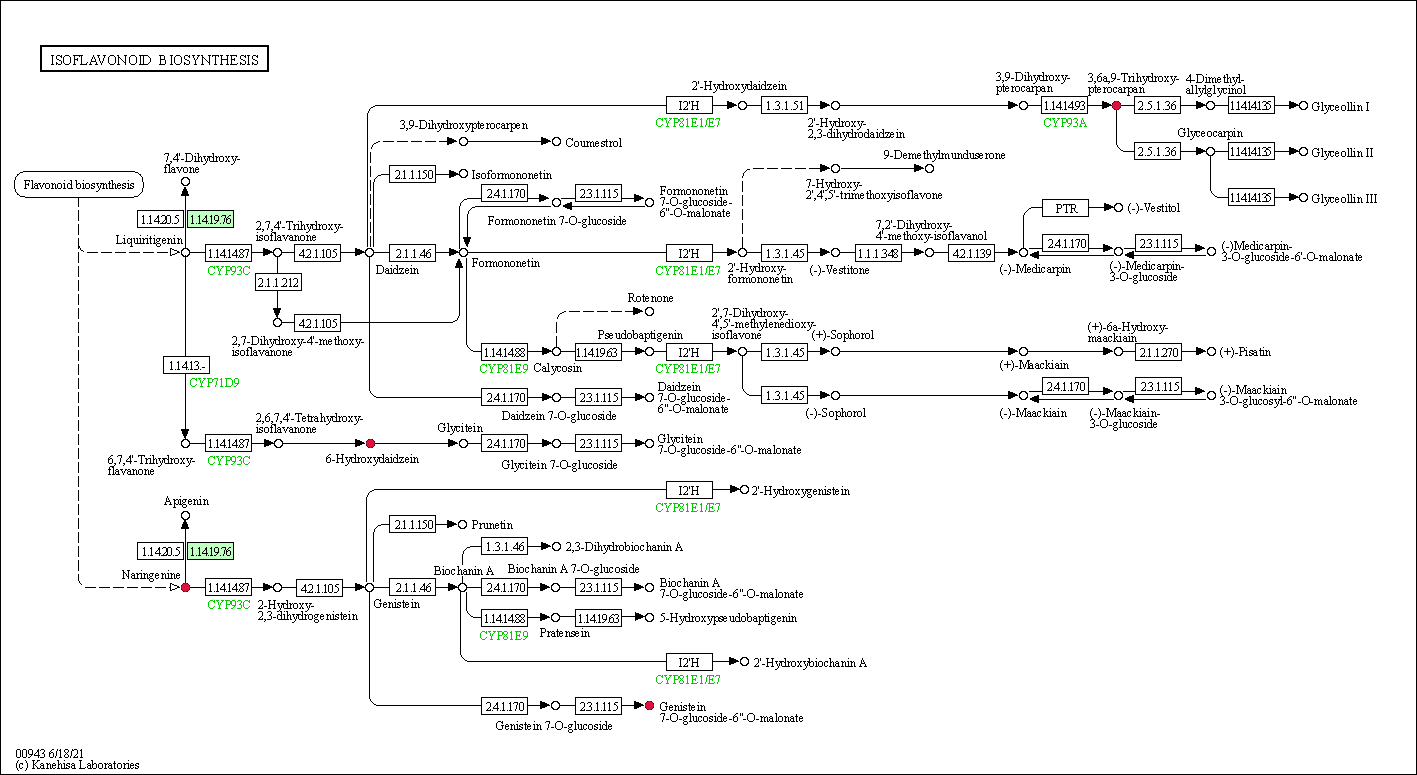

Supplement: Supplementary file 2 [file Image2.png]

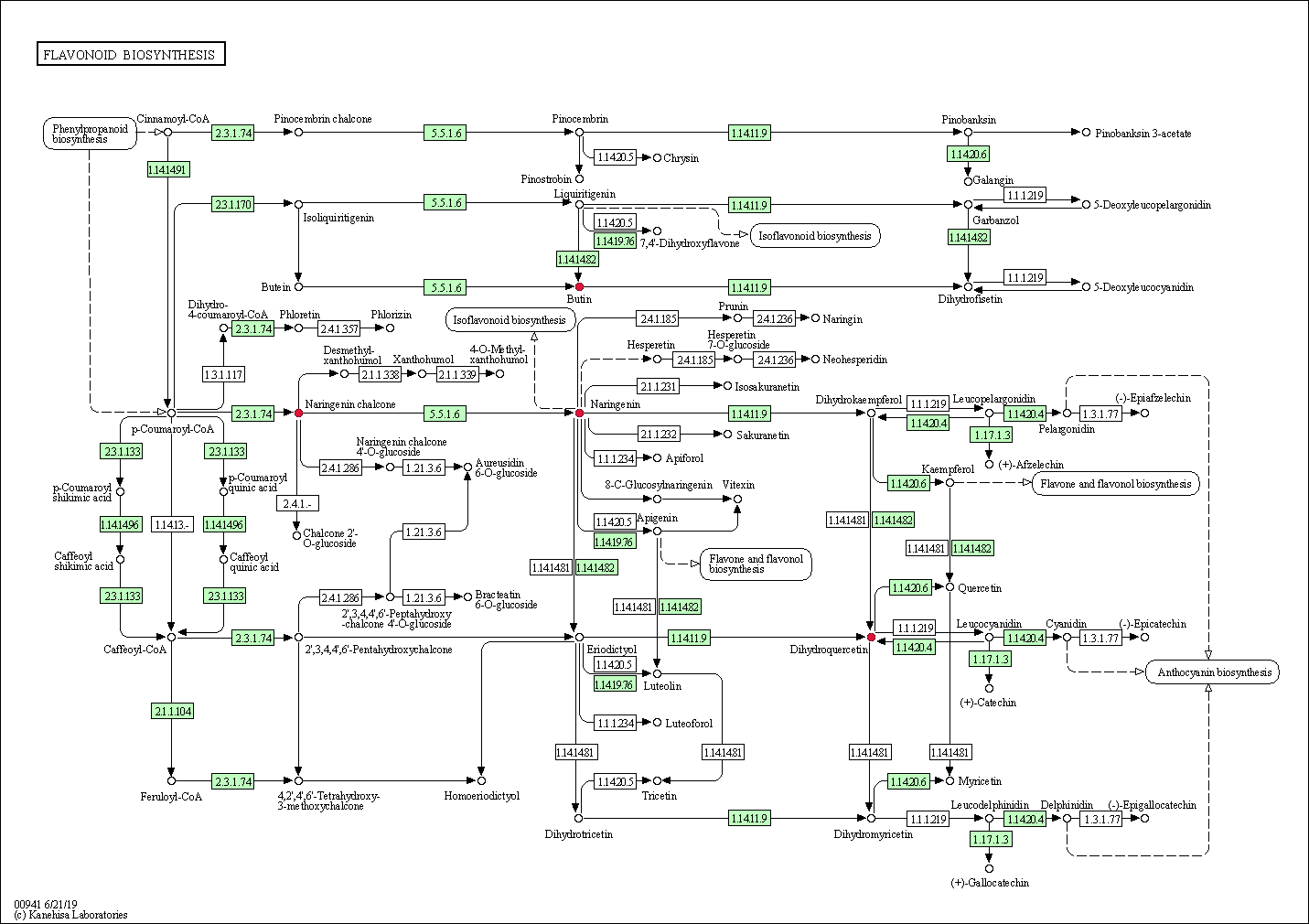

Supplement: Supplementary file 3 [file Image3.png]

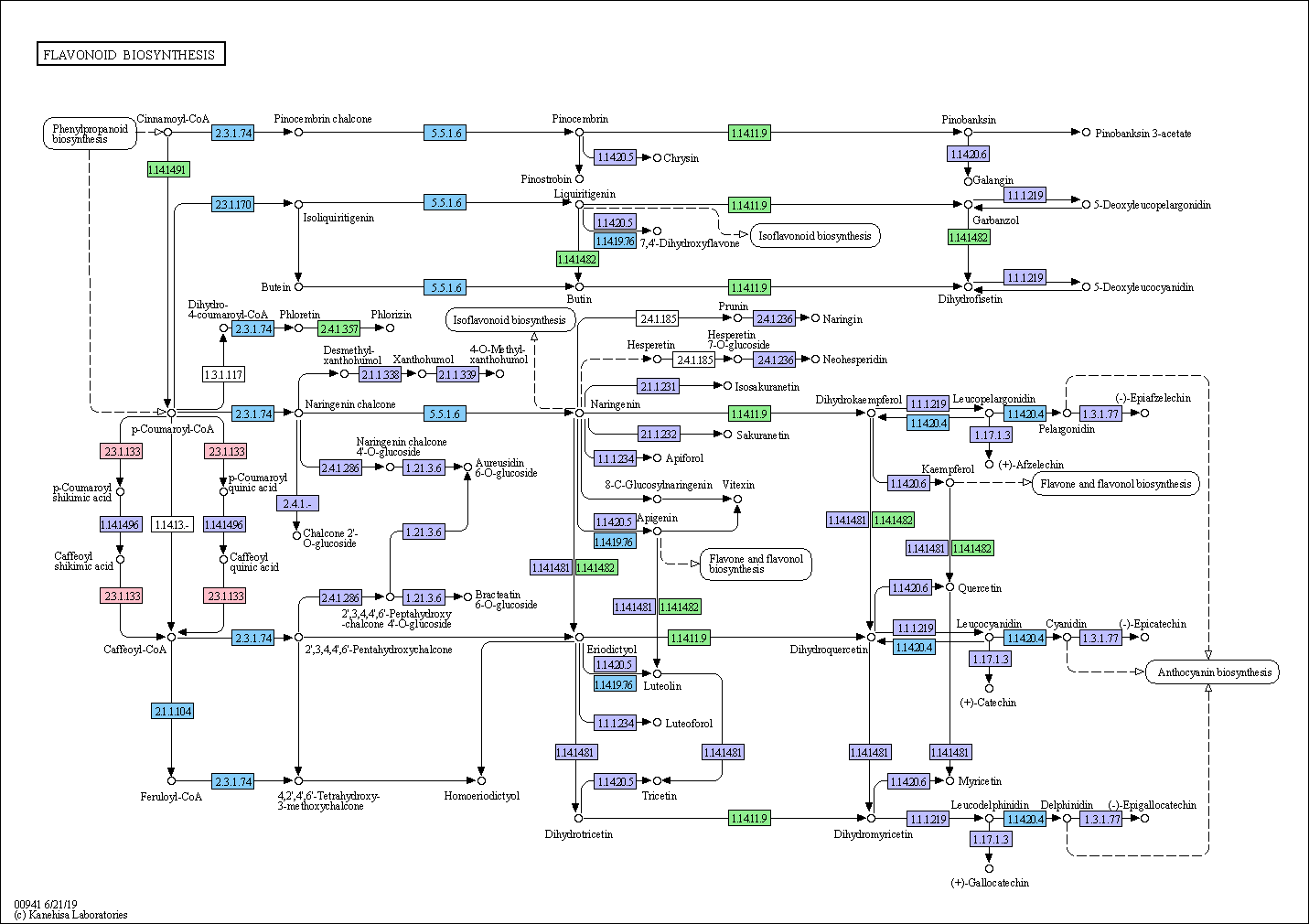

Supplement: Supplementary file 4 [file Image4.png]
