# Supplementary material for: Transcriptomic and metabolomic analyses of three Dendranthema morifolium “Boju” varieties with different flower colors
Source: Front Plant Sci. 2026 Feb 12;16:1690517. doi: 10.3389/fpls.2025.1690517 (PMC12935939; doi:10.3389/fpls.2025.1690517)
Supplement: Supplementary file 6 [file Table2.doc]

S1:

The capitulum of ‘Boju’ in different colors: 1-a. pinkish-white (FB), 1-b. yellowish-white (HB) 1-c. pure yellow (CH).

S2:

Transcriptome sequencing quelity analysis.

S3 and S4 :

The small circles in the metabolic pathway diagram represent metabolites. The metabolites marked in red in the pathway diagram are the up-regulated metabolites detected experimentally, while those marked in blue are the down-regulated metabolites. Yellow indicates both an increase and a decrease.

S5:

In the figure, the boxes represent functional proteins, corresponding to characteristics such as genes, proteins, and microbial functions (when genes and proteins are colored simultaneously, the left side represents genes and the right side represents proteins), and the circles represent metabolites. The blue series indicates a downward adjustment of the difference, the orange series indicates an upward adjustment of the difference, and the yellow series indicates no downward adjustment information. For details, please refer to the legend in the upper left corner. A single omics element is represented by multiple genes in bright blue (RGB (0,255,255)).
